# Income Disparities in Cancer Screening: A Cross-Sectional Study of the Korean National Health and Nutrition Examination Survey, 2013–2019

**DOI:** 10.3389/fpubh.2022.820643

**Published:** 2022-03-17

**Authors:** Vasuki Rajaguru, Tae Hyun Kim, Jaeyong Shin, Sang Gyu Lee

**Affiliations:** ^1^Department of Healthcare Management, Graduate School of Public Health, College of Medicine, Yonsei University, Seoul, South Korea; ^2^Department of Preventive Medicine, College of Medicine, Yonsei University, Seoul, South Korea; ^3^Institute of Health Services Research, Yonsei University, Seoul, South Korea

**Keywords:** cancer, cancer screening, income disparity, public health, health status

## Abstract

**Background:**

Cancer is one of the leading chronic diseases, which causes premature mortality in Korea. Early detection has been reported to be associated with reduced mortality and morbidity. Consistent evidence reports that lower screening rates are associated with socioeconomic-based disparities. This study aimed to examine income-related disparities in cancer screening services and to analyze the association between utilization of cancer screening and individual characteristics, including income levels.

**Methods:**

This study utilized the data from the Korea National Health and Nutrition Examination Survey (KNHANES), a population-based survey from 2013 to 2019. The study population included individuals aged 40 years or over. The variables were socioeconomic characteristics and perceived health status. Household income was categorized into quartiles from Q1 (the lowest income group) to Q4 (the highest income group). Multivariate logistic regression analysis was performed to analyze the association between cancer screening and individual characteristics and household income levels.

**Results:**

There were 20,347 individuals included in this study. Among these, 14,741 (72.4%) had undergone cancer screening. There existed a gap in the utilization of cancer screening between the lowest (Q1) and highest (Q4) income quintiles owing to evident income disparities; Q4 thus had a significantly higher likelihood of undergoing cancer screening than other quintiles. Female sex, university and over education, number of chronic diseases, and private insurance coverage were positively associated with cancer screening (*p* < 0.001).

**Conclusion:**

Our findings suggest that policymakers should develop and design strategies to increase awareness and efforts to improve the education and promotion of cancer screening among lower-income target groups.

## Background

Cancer is a global public health challenge. The cancer incidence and mortality rate are rapidly increasing worldwide; also have been reported in developed countries over the last few decades (1, 2). The incidence of cancer has increased to an epidemic level in South Korea (2). The number of incident cancer cases was estimated at 229,180, of which 27.8% of cancer-related deaths occurred between 2011 and 2016 (3). The cancer-related economic burden was reported exhibited an average 8.9% annual growth rate in 2010 (4), accounting for 0.23% of the national gross domestic product and 1.36% of national healthcare expenditure in 2014 (5) and, in terms of the total cost, $1 was equal to 1,131.52 won in 2015, according to Statistics Korea (6).

Screening is seen by many as a key element in cancer control strategies (7). Cancer could be prevented by screening and early detection; ~50–60% of the cancer cases are detected early with the commonly used, well-organized strategies (8). Regular, population-based cancer screening results in earlier detection and increased survival, and there is evidence that regular risk-appropriate screening may reduce cancer mortality (9, 10).

In Korea, the National Cancer Screening Program (NCSP) has conducted population-based screening since 1999 for providing free screening services (2, 11); it was expanded rapidly for these groups, and this, in turn, increased the scope of screening. National Cancer Screening Program provides free cancer screening services for five common cancers: gastric, liver, colorectal, breast, and cervix to Medical Aid recipients and National Health Insurance beneficiaries in the lower 50% income population (10–12). In addition, special screening is also an opportunistic screening tool in outpatient clinics or private health assessment centers (10). However, in these cases, individuals must pay for opportunistic screening for all types of cancer-related screening procedures.

The literature findings revealed that utilization of cancer screening was associated with socioeconomic factors in Korea (13–20) and other countries (21–24), cognitive factors, and predisposing factors (24–30), health care system factors such as health insurance, and national screening programs (11–14, 25–28, 31). Previous studies have investigated the specific type of cancer related to economic inequality and disparity with different cancers (13–16, 21, 22, 24, 25, 29, 31–34). Differences in cancer screening related to socioeconomic status may contribute to morbidity and mortality variations across Korea (15, 16, 33, 34). Some studies have studied income inequalities that affect the utilization of cancer screening (13, 16–23, 28–32, 35).

Overall the decades, Korea also experienced rapid socioeconomic growth decline not only in the lifestyle changes but also in the wide of income inequality. Therefore, Korea's homogenous sample population analyzes the association between income disparities and various diseases in Korea (13–22, 32). Numerous studies have been reported, by using the different databases to analyze the relationship or factors associated with specific types of cancer screening than overall cancer screening (13–23, 25, 31, 32, 34). However, differences across income groups receiving the screening are still observed, with 41% in the lowest income quartile receiving screening compared with 54 % in the highest in 2012 (30).

Existing studies have been focused on socioeconomic disparity with specific types of cancer screening such as breast (22), cervical or both (13, 14, 22, 24, 32), colorectal (25), gastric (31), and thyroid (13). However, inconsistent results have been reported regarding overall cancer screening. Clarifying these inconclusive results is important because information on income disparity in cancer screening might be useful to identify the individuals at risk of cancer. As there is limited evidence on whether or not the NCSP program contributes to encouraging individuals with lower income to utilize the screening by using the nationally conducted cross-sectional survey. It is unknown whether the current population-based national screening program supports diminishing income-related disparities in cancer screening services (9–12, 16–18, 20). However, as studies cited above, increases in screening rates might not always appreciate equitably across socioeconomic or income status, and not yet been directly investigated so far using 5 years of data from the Korea National Health and Nutrition Examination Survey (KNHANES) after 2014 (6th cycle of the survey), the 8th survey (2019) has recently been reported, and also planned to provide the updated cancer screening trends before the pandemic era. In addition, the NCSP is a nationwide organized program, offering cancer screening for the population aged 40 years or over.

This study aimed to investigate the cancer screening rates and trends from 2013 to 2019 by using the KNHANES data among the population aged 40 years or over, we also assessed the contribution of socio-economic factors, including income-based disparities in the utilization of cancer screening.

## Methods

### Data Source and Population

This study used standardized cross-sectional data from the Korea National Health and Nutrition Examination Survey (KNHANES), which is a population-based survey that provides comprehensive data on health status, healthcare utilization, and socioeconomic status of an entire Korean population (16). It was established in 2007 and the survey has been conducted every year and composed as a cycle, it consists of survey data every 3 years. The KNHANES samples are based on a multi-stage clustered sample of the non-institutionalized Korean population from the household registries. The primary sampling units (PSU) were selected across Korea and the survey weights are provided by adjusting for complex survey designs with post-stratification. We used the 6th, 7th, and 8th surveys (8th survey data are available until 2019) from the KNHANES, over 40 years population are included in this study. It contains questions to obtain basic information on household income and individual characteristics regarding demographics, health status, and lifestyle.

### Variables and Measurement

#### The Dependent Variable

The dependent variable of this study was whether participants had undergone a cancer screening (Question: “Have you undergone cancer screening in past 2 years?”; answer: “Yes” or “No”). The type of cancer screening was classified as (1) free of cost, (2) self-payment, or (3) partial payment screening at comprehensive cancer screening in public, private, or general hospitals.

#### Independent Variables

##### Socioeconomic Characteristics

The demographic characteristics included age, sex, education status, marital status, job, income, residence (based on the participant's residence), and types of insurance. The age group was divided into four groups (40–49, 50–59, 60–69, and ≥70 years) starting from age 40 years, which is the national cancer screening recommendation standard. Marital status was classified as married, single, and divorced/widowed. The education status had four categories: elementary school, middle school, high school, and university and over. The ccupation was classified as “yes” or “no” according to the participant's answer (all types of work or no work, respectively). Residential areas were divided into urban (city or town) and rural (village) areas. The level of household income was calculated by dividing the household monthly income by the square root of the household size (equalized income), and classified into four categories: lowest, middle, high, and highest in the quartile (35). In terms of insurance types, private medical benefit membership was recorded as “yes” or “no” and national health insurance was classified into two groups according to a response of “yes” (employee and family/self-membership) or “no” (no response).

##### Lifestyle and Health-Status Characteristics

Lifestyle and health-related variables included participants' perceived health status, stress, smoking, alcohol consumption, physical activity (moderate), cancer, and chronic diseases. Self-reported health status was classified into three categories: fair (very good/good), moderate, and bad (bad/very bad). In self-perceived stress level, "the participants were asked for their perceived stress levels, and four options were given for response: (1) very much, (2) much, (3) a little, and (4) almost none. They were then divided into two categories; the first two responses were considered as the high-level stress (Yes), and the last two were classified as the low-stress (No). Alcohol consumption was categorized into two groups according to the amount and frequency of alcohol consumed monthly included; (1) “Yes” (high-risk drinkers), defined as those who consume more than seven (for men) or five (for women) drinks on a single time at least once per month; and (2) “No” low-risk drinkers, defined as non-drinkers and those who drink less than once per month (19). Smoking was divided into current (present smoker/sometimes), former (past smoker), and never (lifetime non-smoker). Physical activity was defined as engaging in ≥10 min of physical activity that produces at least slight breathlessness and sweating less than once per week (20), and divided in to two categories that: “Yes” or “No” from the following question: “Do you engage in moderately intense sports, exercise or recreational activities that result in an elevated heart rate?” The presence or absence of cancer was recorded as “Yes” or “No” regarding all types considered among 18 diseases (all types of cancer). Chronic diseases were considered based on the International Classification of Diseases (ICD-10) code with recommended diseases: heart disease, stroke, depression, diabetes, arthritis, etc., and the responses were categorized as “1” (any one disease), “2” (any 2 diseases) or “3” (three and more).

“The study was conducted according to the guidelines of the Declaration of Helsinki and approved by the Institutional Review Board of affiliated University (2021-4-0986). In addition, the KNHANES was approved by the KDCA Institutional Review Board (2018-01-03-P-A) in 2018. All participants provided informed consent to participate in the KNHANES and it was ensured that they remained anonymous.”

### Statistical Analysis

Data analysis was performed in three steps. First, chi-square verification was used to calculate the distribution of each covariate. Second, multivariate logistic regression models were used to determine the odds ratio (OR) and 95% confidence interval (CI) to assess the factors associated with cancer screening. Model 1. age, sex, education, marital status, occupation, residence, smoking, alcohol consumption was assigned as an independent variables, and Model 2. household income, chronic diseases, cancer and types of health insurance focused as an independent variables. Third, a subgroup analysis was performed to analyze the factors associated with cancer screening according to household income, with selected covariates. The differences in the influences of income on cancer screening was analyzed with adjusted odd ratio of covariates were examined by using interaction terms in which income quintiles. Statistical significance was defined as *p* < 0.05 (36, 37). All analyses were performed using SAS version 9.4 (SAS Institute Inc., Cary, NC, USA) (36, 37).

## Results

### Study Population and Distribution of Cancer Screening From 2013 to 2019

**Figure 1:**
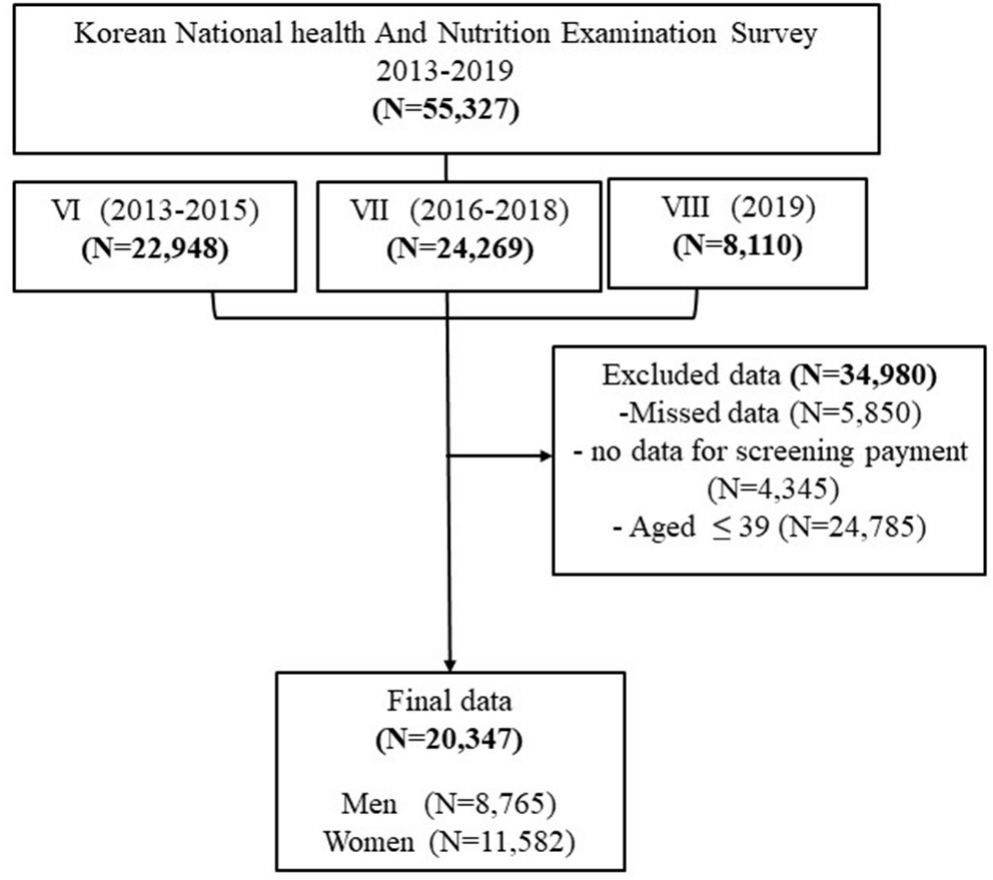
Flow diagrams showing selection of the study population.

**Figure 2:**
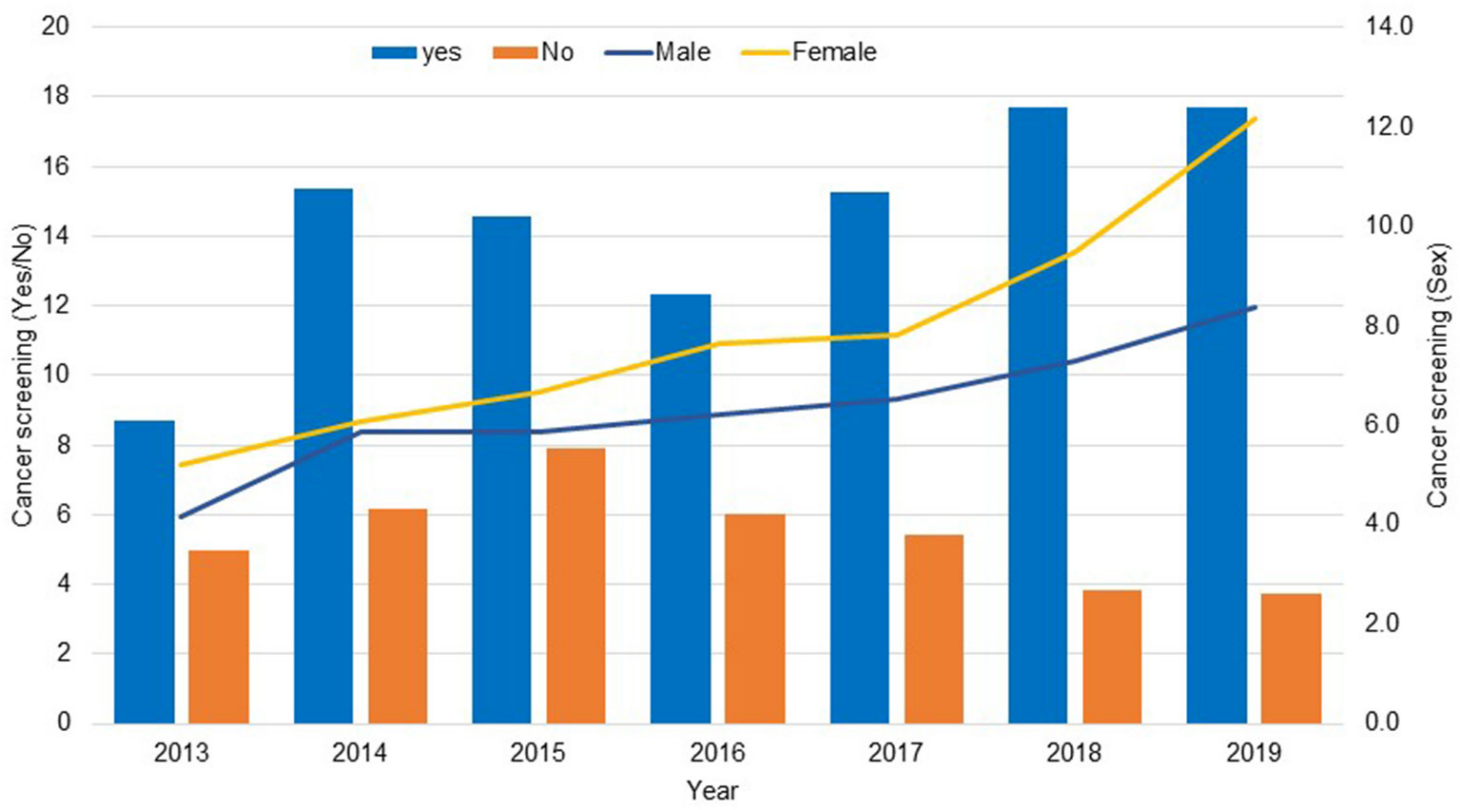
Frequency distribution of cancer screening (Yes/No) and sex based on the KNHANES data 2013–2019.

The total number of households participating in the KNHANES for 2013 through 2019 was 55,327. A total 20,347 male and female participants aged 40 years and older were included in this study (Figure 1). The frequency distribution of cancer screening was gradually incresased in 2019 and then and a lower percentage of participants exposed to cancer screening in 2013 (38%). The number of women who underwent cancer screening was higher than men (55.7 vs. 44.3%, respectively) (Figure 2).

### Chi-Sqaurte Test Analysis of Socioeconomic Characteristics of Cancer Screening

The final data consisted of 20,347 participants aged 40 years and over. Table 1 compares the characteristics of cancer screening. The frequency of cancer screening was 14,741 (72.4%). Regarding socioeconomic characteristics, most participants who underwent cancer screening were women (55.7%) and within the 50–59 years age group (27.8%). Regarding education, most of them had a university or over (33.2%). Regarding marital status, majority of participants were married (96%) and working (91.1%). About 2/3 of the participants were residing in urban areas (78.6%) and most participants had household income in the highest quartile (28.8%). The types of insurance reported included national health (94.4%) and private insurance (70.3%).

**Table 1:** Distribution of cancer screening based on general characteristics of participants.

**Variables**	**Cancer screening**						**χ2**	***p***
	**Total**		**Yes**		**No**			
	***N***	**%**	***N***	**%**	***N***	**%**		
	20,347	100	14,741	72.4	5,606	27.6		
**Sex**								
Male	8,765	43.1	6,526	75	2,239	26	31.079	<0.001
Female	11,582	56.9	8,215	71	3,367	29		
**Age**								
40–49	4,690	23.1	3,040	64.8	1,650	35.2	51.854	<0.001
50–59	5,498	27.0	3,711	67.5	1,787	32.5		
60–69	5,944	29.2	3,456	58.1	2,488	41.9		
≥70	4,215	20.7	2,563	60.8	1,652	39.2		
**Level of education**								
Elementary school	4,182	20.6	1,795	42.9	2,387	57.1	242.231	<0.001
Middle school	4,684	23	4,013	85.7	671	14.3		
High school	5,509	27.1	4,038	73.3	1,471	26.7		
University and over	5,872	28.9	4,795	81.7	1,077	18.3		
**Marital status**								
Married	19,194	94.3	14,151	73.7	5,043	26.3	7.891	<0.001
Single								
Divorced/widowed	1,153	5.7	590	51.2	563	48.8		
**Occupation**								
Yes	18,736	92.1	13,430	71.7	5,306	28.3	103.485	<0.001
No	1,601	7.9	1,301	81.3	300	18.7		
**Residence**								
Urban	15,859	77.9	11,582	73	4,277	27	12.245	<0.001
Rural	4,488	22.1	3,159	70.4	1,329	29.6		
**Household income**								
Q1 (Lowest)	5,191	25.5	3,123	60.2	2,068	39.8	366.378	<0.001
Q2	4,919	24.2	3,399	69.1	1,520	30.9		
Q3	5,108	25.1	3,980	77.9	1,128	22.1		
Q4 (Highest)	5,129	25.2	4,239	82.6	890	17.4		
**National health insurance**								
Yes	17,194	84.5	14,151	82.3	3,043	17.7	220.831	<0.001
No	2,153	10.6	590	27.4	1,563	72.6		
**Private health insurance**								
Yes	13,319	65.5	10,367	77.8	2,952	22.2	560.841	<0.001
No	7,028	34.5	4,374	62.2	2,654	37.8		
**Self-reported health status**								
Fair	3,525	17.3	2,715	77	810	23	24.316	<0.001
Moderate	13,343	65.6	9,607	72	3,736	28		
Bad/poor	3,479	17.1	2,419	69.5	1,060	30.5		
**Self-perceived stress**								
Often	4,259	20.9	2,962	69.5	1,297	30.5	25.952	<0.001
Rarely	16,088	79.1	11,779	73.2	4,309	26.8		
**Smoking**								
Never	1,012	5	661	65.3	351	34.7	186.746	<0.001
Former	14,944	73.4	11,162	74.7	3,782	25.3		
Present	4,391	21.6	2,918	66.5	1,473	33.5		
**Alcohol consumption**								
Yes	3,471	17.1	2,612	75.3	859	24.7	16.484	<0.001
No	16,876	82.9	12,129	71.9	4,747	28.1		
**Physical activity**								
Yes	16,526	81.2	11,997	72.6	4,529	27.4	4.645	<0.001
No	3,821	18.8	2,744	71.8	1,077	28.2		
**Cancer**								
Yes	20,232	99.4	14,741	72.9	5,491	27.1	18.834	<0.001
No	115	0.6	0	0	115	100		
**Chronic disease status**								
1	10,877	53.5	3,063	28.2	7,814	71.8	16.702	<0.001
2	6,884	33.8	1,770	25.7	5,114	74.3		
≥3	2,586	12.7	773	29.9	1,813	70.1		

*“Others” includes never married, separated, and divorced*.

Most participants self-reported fair health status (65.2%), rarely experienced self-perceived stress (79.9%), were former smokers (75.7%), and had habit of alcohol consumption (68.7%). Those who had regular physical activity (81.4%), and more than two chronic diseases (53%) are underwent cancer screening. Chi-square analysis results of comparison between the cancer screening and no screening with demographic variables showed statistically significant differences at *p* < 0.001 level (Table 1).

### Comparison of Types of Cancer Screening Pauyment by Household Income

Table 2 shows cancer screening according to the type and household income. Payment data were not available in 2017 and 2018. Therefore, a total of 13,069 responses were analyzed to determine cancer screening payment types, and most of the participants responded to more than two types of cancer screening. Overall, the findings showed that the percentage of participants free of payment was higher (56.2%) than the percentage of participants with partial payment (45.1%) and self-payment (15.1%). In the case of self-payment cancer screening, the 4th quartile was 6.3%, partial payment (13.8%), and free payment was higher in the lowest group (14.7%). The results were statistically significant between the type of cancer screening payment and household income.

**Table 2:** Frequency distribution of types of cancer screening by household income.

**Cancer screening payment types**	**Total** ***N*** **(%)**		**Household Income**								
			**Q1 (Lowest)**		**Q2**		**Q3**		**Q4 (Highest)**		***p***
	**Yes**	**No**	**Yes**	**No**	**Yes**	**No**	**Yes**	**No**	**Yes**	**No**	
Self-paid	1,977 (15.1)	11,092 (84.9)	240 (1.8)	2,527 (19.3)	408 (3.1)	2,833 (21.7)	500 (3.8)	2,704 (20.7)	829 (6.3)	3,028 (23.2)	<0.0001
Partially paid	5,891 (45.1)	7,178 (54.9)	1,007 (7.7)	1,760 (13.5)	1,491 (11.4)	1,750 (13.4)	1,584 (12.1)	1,620 (12.4)	1,809 (13.8)	2,048 (15.7)	<0.0001
Free of pay	7,343 (56.2)	5,726 (43.8)	1,926 (14.7)	901 (6.9)	1,866 (14.3)	1,315 (10.1)	1,766 (13.5)	1,438 (11)	1,785 (13.7)	2,072 (15.9)	<0.0001

### Multivariate Logistic Regression Analysis of Factors Associated With Cancer Screening and Covariates

Table 3 summarizes the results of the multivariate logistic regression models of factors associated between cancer screening and selected independent variables. In Model 1, differences of cancer screening among male are showed less likelihood of cancer screening compared to women. The age of 50–59 years (OR = 1.07, 95% CI, 1.00–1.11) are showed good proportion than other age groups. The odds ratio of having a university or over (OR = 1.25, 1.02–1.47) was significantly associated with cancer screening rather than having an elementary-level education. In terms of occupation was significantly more likely to have cancer screening (OR = 1.41, 95% CI, 1.15–1.73) than the no occupation. Participants residing in urban areas (OR = 1.11, 95% CI, 1.02–1.19) and not consuming alcohol (OR = 1.24, 95% CI, 1.16–1.30) were more likely to undergo cancer screening than their counterparts.

**Table 3:** Multivariate logistic regression of factors associated with cancer screening.

**Variables**	**Cancer screening (Yes)**			
	Model I^*^	***P***	Mode II^*^	***P***
	**OR (95% CI)**		**OR (95% CI)**	
**Sex**				
Male	0.76 (0.66–0.86)	0.442	0.81 (0.55–1.35)	0.501
Female	1.00		1.00	
**Age**				
40–49	1.00		1.00	
50–59	0.76 (0.74–0.80)	0.251	0.84 (0.80–0.90)	<0.001
60–69	0.90 (0.47–1.74)	0.032	0.96 (0.75–1.11)	0.402
≥70	0.91 (0.86–0.97)	<0.001	0.99 (0.89–1.11)	0.451
**Education**				
Elementary school	1.00		1.00	
Middle school	0.78 (0.74–0.82)	<0.001	0.86 (0.82–0.91)	<0.001
High school	0.88 (0.84–0.93)	<0.001	0.89 (0.86–0.93)	<0.001
University or higher	1.25 (1.02–1.47)	0.004	1.11 (0.99–1.24)	<0.001
**Marital status**				
Married	1.00		1.00	
Others	0.92 (0.87–0.97)	<0.001	1.08 (0.94–1.25)	0.045
**Occupation**				
Yes	1.41 (1.15–1.73)	<0.001	1.27 (0.57–2.84)	0.055
No	1.00		1.00	
**Residence**				
Urban	1.11 (1.02–1.19)	<0.001	1.12 (1.01–1.18)	0.431
Rural	1.00		1.00	
**Smoking**				
Never	1.00		1.00	
Former	0.86 (0.68–1.07)	0.612	0.78 (0.49–1.28)	0.324
Present	0.80 (0.71–0.93)	0.004	0.89 (0.78–1.07)	0.582
**Alcohol drinking**				
No	1.24 (1.16–1.30)	<0.001	1.55 (1.42–1.71)	<0.001
Yes	1.00		1.00	
**Chronic disease status**				
1			1.00	
2			1.14 (1.02–1.30)	0.003
≥3			1.09 (1.02–1.16)	<0.001
**National health insurance**				
No			1.00	
Yes			1.45 (0.89–2.22)	0.051
**Private health insurance**				
No			1.00	<0.001
Yes			2.73 (1.50–4.94)	
**Household income**				
Q1 (Lowest)			1.00	
Q2			1.01 (0.69–1.48)	0.023
Q3			3.86 (1.64–9.09)	<0.001
Q4 (Highest)			4.07 (1.63–10.13)	<0.001
Likelihood ratio test: *p* < 0.05 Hosmer Lemeshow *p* = 0.381 > 0.05		Likelihood ratio test test: *p* < 0.05 Hosmer Lemeshow *p* = 0.293 > 0.05		

* *Hosmer-Lemeshow or likelihood ratio test; OR, odds ratio; CI, confidence interval*.

*Model 1: Age, sex, education, marital status, occupation, residence, smoking, alcohol consumption with cancer screening “Yes”*.

*Model 2: Age, sex, education, marital status, occupation, residence, smoking, alcohol consumption, chronic diseases, health insurance, and household income with cancer screening “Yes”*.

After adjusting for age, sex, Age, sex, education, marital status, occupation, residence, smoking, alcohol consumption, chronic diseases, health insurance, and household income in model 2, participants with two (OR = 1.14, 95% CI, 1.02–1.30) and ≥3 (OR = 1.06, 95% CI, 1.03–1.16) comorbidity were more likely to undergo cancer screening than individuals with 1 condition. Those who had private health insurance (aOR = 2.73, 95% CI, 1.50–4.94) were more likely to undergo cancer screening than those with no private health insurance. The participants in Q3 (OR = 3.86, 95% CI, 1.64–9.09) and Q4 (OR = 4.07, 95% CI, 1.63–10.13) were more likely to undergo cancer screening than those in Q1. As a result of the Likelihood ratio test, at least one of the independent variables used in the analysis was significant by rejecting the null hypothesis with *p* < 0.05 in all models. The *p*-value of Hosmer and Lemeshow goodness of-fit test logistic regression model showed >0.05, therefore, it can be confirmed that the model was fit to the data well (Table 3).

### Subgroup Analysis of Household Income and Selected Covariates

The subgroup analysis of household income is presented in Table 4. Overall, we observed income-related disparities in cancer screening, i.e., the highest income quintile underwent more cancer screening than the lowest income quintile. In household income group Q4, women (aOR = 1.38, 95% CI, 1.24–1.54), individuals aged 40–49 years (aOR = 1.77, 95% CI, 1.08–2.92), and individuals with private insurance (aOR = 3.06, 95% CI, 1.60–5.13) were more likely to undergo cancer screening (*p* < 0.001) than those of the Q1 household income group. The highest income group of Q4 comprising participants with university and higher education and chronic diseases showed a higher aOR for undergoing cancer screening than those who had low household income, elementary-level education, and one chronic disease (Table 4).

**Table 4:** Subgroup analysis of associations between cancer screening and household income.

**Variables**	**Cancer screening**			
	**Q1 (Lowest)**	**Q2**	**Q3**	**Q4 (Highest)**
		**aOR (95% CI)**	**aOR (95% CI)**	**aOR (95% CI)**
**Sex**				
Male	1.00	0.56 (0.45–0.7)	0.99 (0.94–1.22)	1.05 (1.03–1.08)
Female	1.00	0.96 (0.92–1.01)	1.31 (1.21–1.38)	1.38 (1.24–1.54)
**Age (Years)**				
40–49	1.00	0.55 (0.32–0.98)	0.64 (0.10–3.96)	1.77 (1.08–2.92)
50–59	1.00	0.43 (0.35–0.53)	0.92 (0.88–1.01)	1.29 (1.21–1.37)
60–69	1.00	0.87 (0.64–1.20)	0.89 (0.45–1.71)	0.90 (0.71–1.11)
≥70	1.00	0.83 (0.70–4.81)	0.98 (0.95–1.02)	1.05 (1.00–1.10)
**Education**				
Elementary school	1.00	1.04 (1.00–1.09)	1.24 (1.20–1.30)	1.32 (1.15–1.53)
Middle school	1.00	1.25 (1.11–1.41)	1.40 (1.21–1.60)	1.41 (1.32–1.54)
High school	1.00	1.28 (1.14–1.43)	1.58 (1.41–1.76)	1.66 (1.44–1.91)
university or over	1.00	1.66 (1.48–1.86)	1.85 (1.71–3.82)	1.91 (1.66–2.20)
**Cancer**				
No	1.00	0.74(0.41–1.33)	0.65 (0.38–1.12)	0.98 (0.81–1.7)
Yes	1.00	0.88 (0.84–0.93)	1.37 (0.58–3.23)	2.32 (1.07–5.02)
**Chronic disease status**				
1	1.00	1.06 (1.00–1.13)	1.20 (1.04–1.38)	1.11 (1.01–1.13)
2	1.00	1.04 (1.03–1.06)	1.29 (1.14–1.46)	1.75 (1.55–1.98)
≥3	1.00	1.36 (1.08–1.71)	1.47 (1.33–1.61)	1.90 (1.85–1.96)
**Health Insurance**				
National	1.00	1.12 (0.95–1.32)	1.50 (1.33–1.69)	1.76 (1.05–2.96)
Private	1.00	1.20 (1.04–1.38)	2.75 (2.52–3.01)	3.06 (1.60–5.13)

*aOR, Adjusted odds ratio; CI, confidence interval*.

*All variables are adjusted for age, sex, education, marital status, residence, job, smoking, perceived health status, stress level, alcohol consumption, physical activity, no chronic diseases, and type of health insurance*.

## Discussion

This study aimed to analyze income disparities in the utilization of cancer screening services by using data from the KHNANES from to 2013–2019 and identified associations of various socio-demographic and health status characteristics. The study findings suggest that income disparities in cancer screening are strongly associated with education, the number of chronic diseases, and private health insurance membership.

Using a large sample of nationwide data, this study found that socioeconomic status and lifestyle characteristics were associated with cancer screening utilization. Our findings revealed that, women have higher screening rates than men. This result emphasizes the need to generate more awareness about cancer screening among male participants. This may be explained by the fact that women have more routine health visits such gynecological visits then men. The age group of 50–59 years, highest education status, non-alcohol users, and urban residence were significantly associated with cancer screening participation.

Cancer screening showed an increasing trend among the age group of 50–59 years; it is widely accepted that middle aged individuals may be more health conscious than older individuals (14, 22, 25–27, 31). However, household work, working pressure, and time affect the other age groups' access to health service even though the services are free, making the population more vulnerable to preventable diseases (12, 13, 23, 26–28). Awareness about cancer screening services might change the attitude of older adults to utilize the service.

Regarding lifestyle, those who had university and higher education status, resided in an urban area, did not consume alcohol, never smoked, and exercised regularly had higher cancer screening utilization. According to the review of previous studies on factors related to cancer screening, most studies reported that drinking, exercise, and smoking did not have a significant correlation with cancer screening (14, 21, 22, 25, 31, 32). However, in some studies, alcohol, smoking, and exercise habits affected early cancer screening. It has been suggested that there is a significant relationship among these factors toward utilization of cancer screening (14, 25–28, 31, 32). Therefore, necessary action should be taken regarding health awareness and follow-up behaviors toward primary prevention with health care access and screening participation.

When an individual had to bear the complete expense to undergo cancer screening, it was found that Q4 had higher screening participation than Q1, evidently because those with lower incomes could not afford cancer screening. Therefore, individuals' socioeconomic condition, which determines their ability to pay for such care services, was considered a specific cause of poor cancer screening participation.

Regarding income, Q1 (lowest) was negatively associated with selected variables. According to the literature, there was no significant association between utilization of cancer screening and age, private insurance, smoking, moderate-intensity physical activity, and the presence or absence of cancer (13, 21, 27, 32). Sex, private insurance, and number of chronic diseases had a significant effect on the use of cancer screening in the highest income group. Overall, it was found that there are many significant variables affecting the use of cancer screening in the low-income group. These results are supported by findings in the literature, especially that participants in the lowest income group are less likely to utilize cancer screening services (12, 15, 16, 28, 29, 32, 34). It is necessary to raise awareness, develop education-related health behavior, and strengthen screening program recommended to motivate cancer screening in the low-income group.

Previous studies notified that barriers hindering participation in NCSP include less trust in these programs and a lack of awareness of the existence and importance of it (15, 17, 30, 35), since participants believed that the free screening services were of low quality (19, 20, 23, 29, 35). In addition, regarding utilization of cancer screening, there was a lower association among participants who did not have private insurance. In Korea, private insurance is not mandatory for all, and individuals can voluntarily enroll in private insurance in addition to the national health insurance (3, 7–9, 30). However, private insurance covers additional payment, which is not paid by the National Health Insurance. People with high health consciousness or with a high number of chronic conditions may be more likely to have private insurance such as covering cancer screening.

In Korea, NHI enrollment is compulsory for public officials and private school faculty members. An estimated 3–5% of people below the poverty line who cannot afford health insurance premiums are covered by Medicaid (Private insurance) (10, 33, 34). Although the NHI and Medicaid virtually made a guarantee to access universal health insurance coverage, low benefit levels and high out-of-pocket costs-imposed limits on the health services received by some beneficiaries, notably those in lower income brackets (13, 16, 19, 29). In our study, low-income participants had lower prevalence of cancer screening, similar to previous studies (13, 15, 16, 29, 33, 34). This might be due to increased concerns about health expenditure by low-income groups, which might affect utilization of health services such as cancer screening.

Furthermore, our findings suggest that socioeconomic status is a very important factor for providing equal access to prevention-oriented primary care. It is necessary to develop awareness-related interventions at the national and regional levels to reduce income disparities in cancer screening utilization. Our findings are similar to previous studies suggesting that income disparity impacts cancer screening positively (13, 14, 16, 17, 20–23, 29, 32). In addition, cancer screening was highly associated with education level, number of chronic diseases, and private insurance coverage (17, 24, 30, 33, 35). The findings revealed that, the causes of less cancer screening utilization is as an important public health challenge. To increase the proportion of older adults that implement regular preventive measures, it could be useful to improve population-based screening services.

The strength of our study lies on the large sample size and the population based self-reported survey data, and this study focused on income disparities with recently available secondary data. There are some policy implications based on the study findings are as follows: the intension of the cancer screening participation to be increased from the middle-aged adults, it could be viewed as a good impact factor of routine screening behavior. The healthcare professionals or policy administrators need to identify and providing the counseling about screening and early detection of cancer according to socio-economic status also important to reduce the income-based disparities in cancer screening. Cancer screening showed differences based on demographic characteristics such as education level, household income, occupation, and perceived health status, it is recommended to expand the scope of free screening for most common cancers and create an environment and individual general characteristics that would make it easier on participation of the people in cancer screening, such as providing paid vacation time, to resolve this income-based disparities. This study recommended to revise the cancer screening duration from “two years to one year” as a benchmark to determine whether the respondents undergone cancer screening.

Our results should be interpreted with reasonable limitations. First, cancer screening responses were self-reported and may not be accurate in determining type of cancer screening based on self, partial, and free payment. Therefore, we did not perform logistic regression analysis based on the type of cancer screening. However, literature reporting the association between cancer screening and household income is most likely based on the self-reported responses, which may have influenced the results. Second, Our study included chronic conditions but did not draw conclusions on the severity of the disease or types of cancer. Therefore, it is considered that vulnerabilities in utilizing cancer screening arise as a result of income disparity. Third, this study considered self-perceived stress level by two categories, and it was assessed by the question addressed in KNHANES data. Since the responses are based on respondent's self-report, this may have been affected the recall bias. Therefore, future studies could focus on standardized stress assessment tool such as DAAS-21, which is a set of three scales and more useful to consider even small changes in symptoms, rather than focusing on categorical differences.

## Conclusions

In the present study, we found an association between the utilization of cancer screening and the household income group. There is a need to develop specific policies for the expansion of public screening programs for the low-income class, to provide more efficient early cancer screening. It was reported that there were also differences in factors associated with the use of cancer screening according to different categorical levels. We also found that the middle-aged group was a vulnerable group that was more likely to not utilize cancer screening than the older adult group. Cancer is widespread among middle-aged individuals >45 years, and it is a serious public health issue. Therefore, it is necessary to make more intention among middle-aged adults to participate in the cancer screening than older adults. Furthermore, attention and effective collaborative efforts by policymakers, health plan administrators, third-party payers, and healthcare providers are needed. In addition, there is a need to develop specific policies for the expansion of public screening programmes for the low-income class to provide more efficiency to encourage regular early cancer screening, especially in the middle-aged group. In the future, research on related factors will be necessary through analysis of the current use of cancer screening programs according to household income level by specific cancer type among middle-aged adults by using the National Health Insurance Service data.

To mitigate potential policy implications, we have developed the population-based cancer indicators and monitoring (38), under the National Cancer Center collboration project, Korea, which included cancer screening and early detection in order to find the community-based (socioeconomic status) vulnerable risk population. In addition, the planned methods (e.g., searching references lists, panel discussion, model development, independent data extraction or quality checks) are consistent with the highest standards for evidence synthesis. This study believed that the planned methods will identify and provide a rigorous evaluation of the cancer screening-related indicators and policy guidelines.

The recommended policy implications have been divided into population-based screening (38) and opportunistic screening (39) based on evidence of existing research. Opportunistic cancer screening programs differ in terms of the cancers screened, the duration between screenings, and the specific cancer type, based on individual decisions or recommended by the health care providers. The national policymakers continue to refine the research with an eye toward improving policies and appraise the evidence for cancer screening based on developed guidelines. We hope that our population-based cancer screening guidelines and opportunistic cancer screening will be used directly or indirectly to other countries or regions interested according to their organizational and healthcare pattern to improve the cancer screening trends among the entire population.

## Data Availability Statement

The datasets presented in this study can be found in online repositories. The names of the repository/repositories and accession number(s) can be found below: https://knhanes.kdca.go.kr/knhanes/sub03/sub03_01.do.

## Ethics Statement

The studies involving human participants were reviewed and approved by KNHANES was approved by the KDCA Institutional Review Board (2018-01-03-P-A) in 2018. Written informed consent for participation was not required for this study in accordance with the national legislation and the institutional requirements.

## Author Contributions

VR, JS, SL, and THK: conceptualization. SL and JS: methodology. VR: data collection and investigation. VR and JS: writing—original draft preparation. SL and THK: writing. All authors read and approved the final manuscript.

## Conflict of Interest

The authors declare that the research was conducted in the absence of any commercial or financial relationships that could be construed as a potential conflict of interest.

## Publisher's Note

All claims expressed in this article are solely those of the authors and do not necessarily represent those of their affiliated organizations, or those of the publisher, the editors and the reviewers. Any product that may be evaluated in this article, or claim that may be made by its manufacturer, is not guaranteed or endorsed by the publisher.
